# Supplementary material for: Photoemission-based microelectronic devices
Source: Nat Commun. 2016 Nov 4;7:13399. doi: 10.1038/ncomms13399 (PMC5097168; doi:10.1038/ncomms13399)
Supplement: Supplementary Information — Supplementary Figures 1-7 [file ncomms13399-s1.pdf]

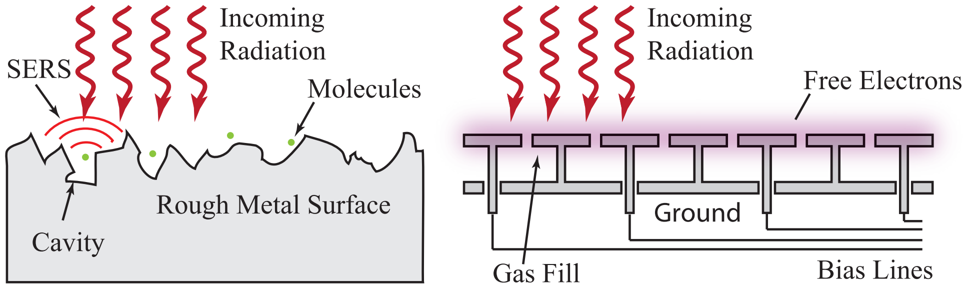

(a) (b)

Supplementary Figure 1. **Surface enhanced Raman spectroscopy (SERS) inspiration**. a) In SERS, the laser-molecule interaction increases due to the surface roughness. b) The proposed electro-optical excitation concept is to activate the photoemission-based device using optical power pumping a designed resonant surface. The bias lines further reduce optical power requirements and provide photonic/electronic controllability.

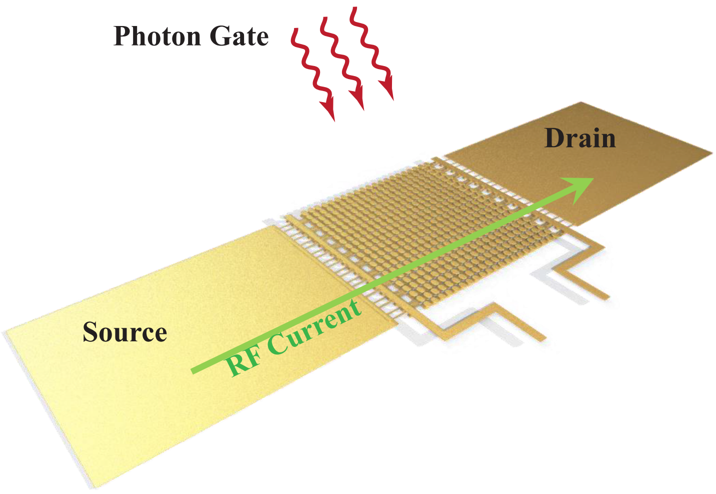

Supplementary Figure 2. **Photoemission-based transistor.** Biased resonant inclusions under illumination by a wavelength-tuned CW laser emit electrons. The free electrons can be manipulated electrically by proper applied voltages. The bias signal along with the incoming laser beam control the conductivity between source and drain.

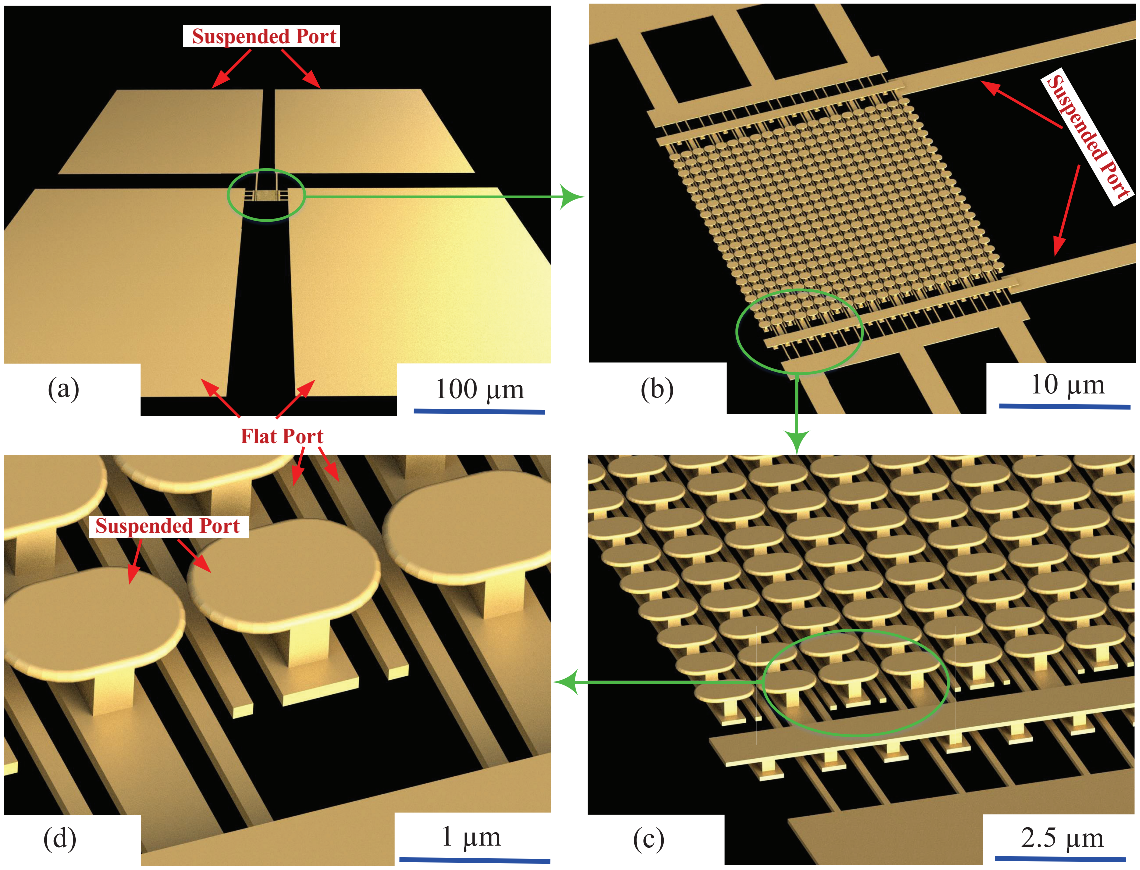

Supplementary Figure 3. **The designed device with two electrical ports.** a) Four 250 µm square pads are used for wire-bonding the device. b) The 20 by 20 unit cells resonant surface. c) Mushroom rows are to be biased with alternating polarities, using the two airbridges on the sides, to form the suspended port. d)The parallel strips on the substrate, below the gaps between the mushrooms, form the flat port.

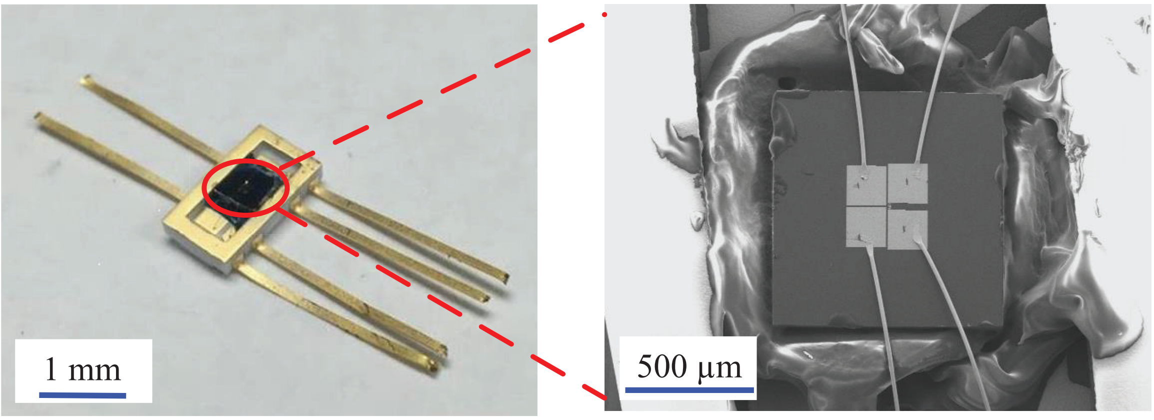

(a) (b)

Supplementary Figure 4. **A device ready for test.** a) A device installed and wire-bonded in the package. b) The SEM picture of the wirebonded device. The fabricated devices were packaged (using standard dual in-line packages) and wire-bonded (using a ball bonder). The packages are ordered from Spectrum Semiconductor Materials Inc. (Part. no. CCF00604). As a confirming experiment, the resonant surface was removed from the design, and a device was fabricated consisting of only the wire bonding square pads. Assuming that the pads are smooth enough to prevent field enhancement, the only conductivity contribution in this device is silicon absorption and temperature rise. The I-V curve of this device showed a negligible change in the conductivity compared to the photoemission-based device.

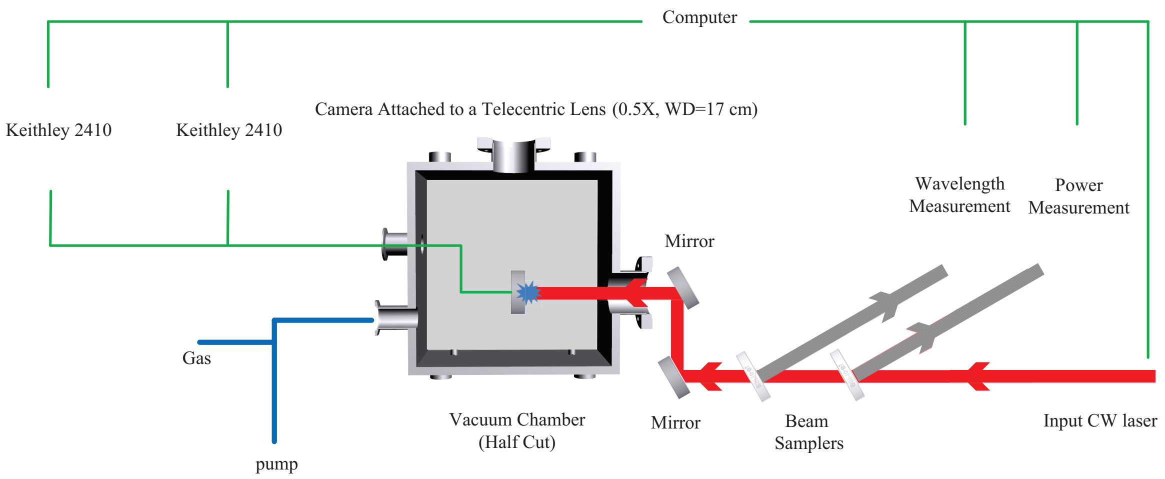

Supplementary Figure 5. **The measurement setup.** Coaxial cables and connectors were used to feed the device inside the vacuum chamber. Use of coaxial cables prevented any plasma formation around the cables, even with applied high voltages.

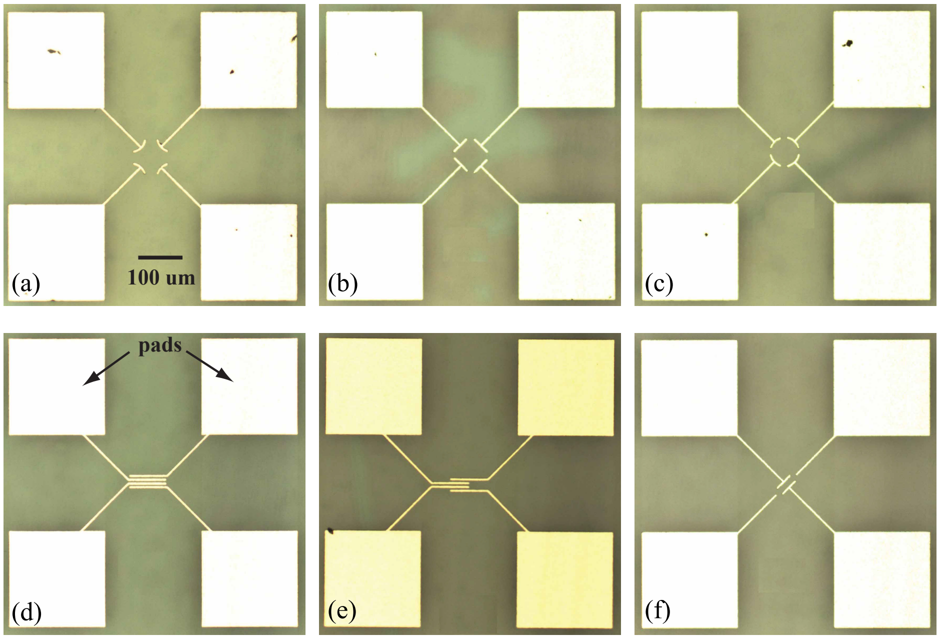

Supplementary Figure 6. **Fabricated non-resonant flat structures on quartz substrate.** a-f) Different orientations and gap spacing between the electrodes without any resonant structure. To confirm the significant effect of the resonant surface, these non-resonant structures (simple flat structures) were fabricated on quartz. None of these structures showed noticeable conductivity change in response to the laser illumination. The measured currents for all of these structures were less than 100 nA even with applied voltages above 100 V on either ports and with large laser illuminations greater than $1\,\mathrm{W\,mm^{-2}}$. Quartz was chosen as the substrate for these flat structures to remove any possible substrate contributions in current measurements. We also tried to fabricate our resonant devices on quartz, but their fabrication was quite challenging since the pattern was too dense and the charge buildup on the isolator substrate distorted the Ebeam lithography repeatedly. Using conductive polymers as the charge dissipator layer did not solve the problem either.

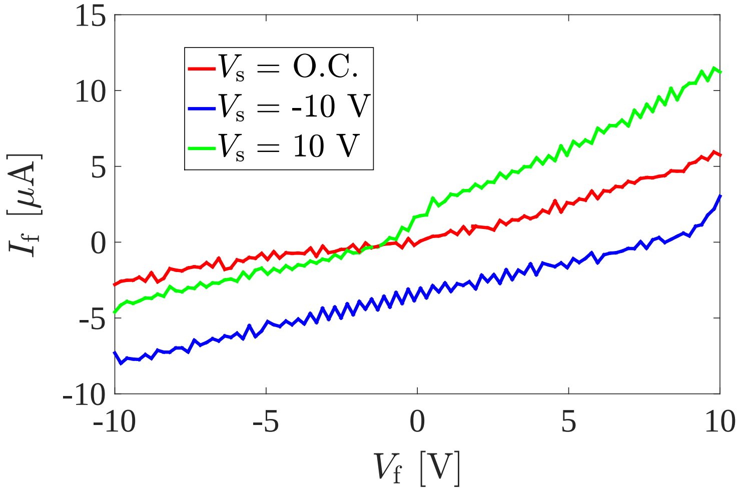

Supplementary Figure 7. **I-V curves of the flat port without the laser illumination.** Current (I_f) versus voltage ((V_f)) of the flat port (of a device fabricated on Si/SiO_2 substrate) with different suspended port excitations (V_s) and without the laser illumination. O.C. indicates open circuit. The conductivity change of the dark device is much smaller than the activated device. The pressure was 0.1 mTorr. The bandgap of SiO_2 is larger than the photon energy at $\lambda = 785$ nm and therefore its resistance does not change with the laser illumination unless the laser causes a change in the temperature, which does not happen during our experiments (we did not use higher laser intensities for this reason). However, the bandgap of silicon is smaller than the photon energy at $\lambda = 785$ nm, and the laser can change its conductivity. If there is a leakage current through the SiO_2 layer and the silicon substrate, some portion of the conductivity change in our experiments could be due to the silicon contribution. We performed a few experiments to gather enough evidence that the photoemission/field emission is dominant in our device. As the simplest experiment, the illumination wavelength on the resonant surface was set to $\lambda = 1050$ nm, at which photons do not have enough energy for coupling to electrons in silicon. The results showed a strong conductivity change (at $\lambda = 1050$ nm, the resonant structure still has enough field enhancement to emit electrons). This was strong evidence that silicon absorption was not important at 785 nm.
